# The Impact of Intersectional Identities on Older People With HIV

**DOI:** 10.1093/geroni/igaa057.2560

**Published:** 2020-12-16

**Authors:** Mark Brennan-Ing, Charles Emlet

## Abstract

Kimberlé Crenshaw introduced the term “intersectionality” in the late 1980s to highlight the experience discrimination and marginalization of Black and African-American women originating from the confluence of their racial/ethnic and gender identities. Since that time the focus on intersectionality has broadened to consider other communities and individuals who may have multiple stigmatized and discredited identities, including older people with HIV (PWH). For example, Porter and Brennan-Ing described the “Five Corners” model as the intersection of ageism, racism, classism, sexism, and HIV stigma for older transgender and gender non-conforming PWH. HIV disproportionately affects marginalized communities (e.g., racial/ethnic and sexual minorities). Thus, for older PWH it is important to consider how HIV stigma may intersect with other marginalized identities and impact physical and psychological well-being. The first paper in this session examines how the intersection of HIV serostatus, gay identity, and age complicates identity disclosure, leading to social isolation and interference with care planning. The second paper describes how intersectional identities among older PWH interfere with access to mental health services in a population that is disproportionately affected by depression and PTSD. Our third paper examines the role of race, education, and behavioral health in neurocognitive functioning among a diverse sample of older HIV+ gay and bisexual men. Our last paper examines neurocognitive functioning among older Latinx PWH, finding that sexual and gender minorities were at greater risk for impairment. Implications of these findings for research and programming that accounts for the effects of intersectionality among older PWH will be discussed.

